# Miquelianin inhibits IAV infection via the MAPK signaling pathway both *in vitro and in vivo*

**DOI:** 10.3389/fimmu.2025.1532336

**Published:** 2025-03-17

**Authors:** He Li, Beilei Shen, Yan Bi, Yan Sun, Shijun Zhang, Kun Xue, Qiuyue Wang, Bingshuo Qian, Junkui Zhang, Lingjun Fan, Zhengyuan Fang, Tiecheng Wang, Yuwei Gao, Donghui Yue

**Affiliations:** ^1^ College of Traditional Chinese Medicine, Changchun University of Chinese Medicine, Changchun, China; ^2^ Changchun Veterinary Research Institute, Chinese Academy of Agricultural Sciences, State Key Laboratory of Pathogen and Biosecurity, Key Laboratory of Jilin Province for Zoonosis Prevention and Control, Changchun, China; ^3^ College of Veterinary Medicine, Shanxi Agricultural University, Jinzhong, China; ^4^ College of Traditional Chinese Medicine, Hainan Medical University, Haikou, Hainan, China; ^5^ School of Pharmacy, Henan University, Kaifeng, China; ^6^ Engineering Research Center of Glycoconjugates, Ministry of Education, Jilin Provincial Key Laboratory of Chemistry and Biology of Changbai Mountain Natural Drugs, School of Life Sciences, Northeast Normal University, Changchun, China

**Keywords:** influenza A virus, flavonoids, miquelianin, network pharmacology, MAPK signaling pathway

## Abstract

**Background:**

Influenza is an acute respiratory infectious disease primarily transmitted through airborne droplets. The prevalence and spread of influenza viruses have significant impacts on global economic development and public health. Current prevention and control strategies for influenza virus infections mainly rely on vaccines and antiviral drugs. However, vaccine efficacy is limited by the antigenic drift and mutation characteristics of influenza viruses, while antiviral drug resistance is increasingly prevalent. Therefore, there is an urgent need for the development of novel antiviral agents. Flavonoids, widely distributed in plants, possess various potent biological properties, including antioxidant, anti-inflammatory, antibacterial, and anticancer activities, which contribute to the management and prevention of numerous diseases. This study aims to investigate the *in vitro* and *in vivo* anti-influenza A virus activity of quercetin, taxifolin, and miquelianin, as well as their underlying.

**Methods:**

*In vitro* infection model (MDCK cells) and mouse lethal infection model of Infuenza A virus were used to evaluate the antiviral activity of quercetin, taxifolin and miquelianin. Subsequently, we applied network pharmacology to elucidate the mechanism of action and validate the findings for miquelianin.

**Results:**

Miquelianin effectively inhibits the replication of H1N1-UI182 both *in vitro* and *in vivo* and provides protection against lethal H1N1-UI182 infection in mice. Compared to virus-infected controls, miquelianin reduces lung injury. Furthermore, by inhibiting the MAPK signaling pathway, miquelianin prevents the overproduction of cytokines, such as IL-6 and IL-1β, induced by viral infection, thereby alleviating inflammatory responses.

**Conclusion:**

Miquelianin is a monomer extracted from traditional Chinese medicine, exhibiting inhibitory effects on H1N1-UI182 replication and lung injury mitigation.

## 1 Introduction

Influenza is an acute respiratory infectious disease primarily caused by influenza viruses, which belong to the family Orthomyxoviridae. This family consists of negative-sense RNA viruses and is further subdivided into four types: A, B, C, and D. Influenza A virus (IAV) can be differentiated into many subtypes based on two surface antigens: hemagglutinin and neuraminidase. Common subtypes include H1N1 and H3N2 in the human populations (1). Influenza B virus (IBV) is further categorized into the Yamagata and Victoria subtypes (2). IAV and IBV are primarily responsible for seasonal influenza outbreaks. According to global epidemiological data, seasonal influenza infects tens of millions of people annually, resulting in between 250,000 and 300,000 deaths and 3,000,000 severe cases (3). While most influenza patients recover within a week, vulnerable populations—such as the elderly (4), infants (5), obese individuals (6), and those with pre-existing health conditions (7)—are at higher risk of severe complications like pneumonia, myocarditis, and encephalitis. Current influenza control measures primarily involve vaccination and pharmacotherapy, including amantadines and neuraminidase inhibitors. However, due to the influenza virus’s ability to rapidly develop resistance to antiviral drugs through antigenic drift or shift (8), there is an urgent need for the development of novel antiviral agents (9).

**Figure 1:**
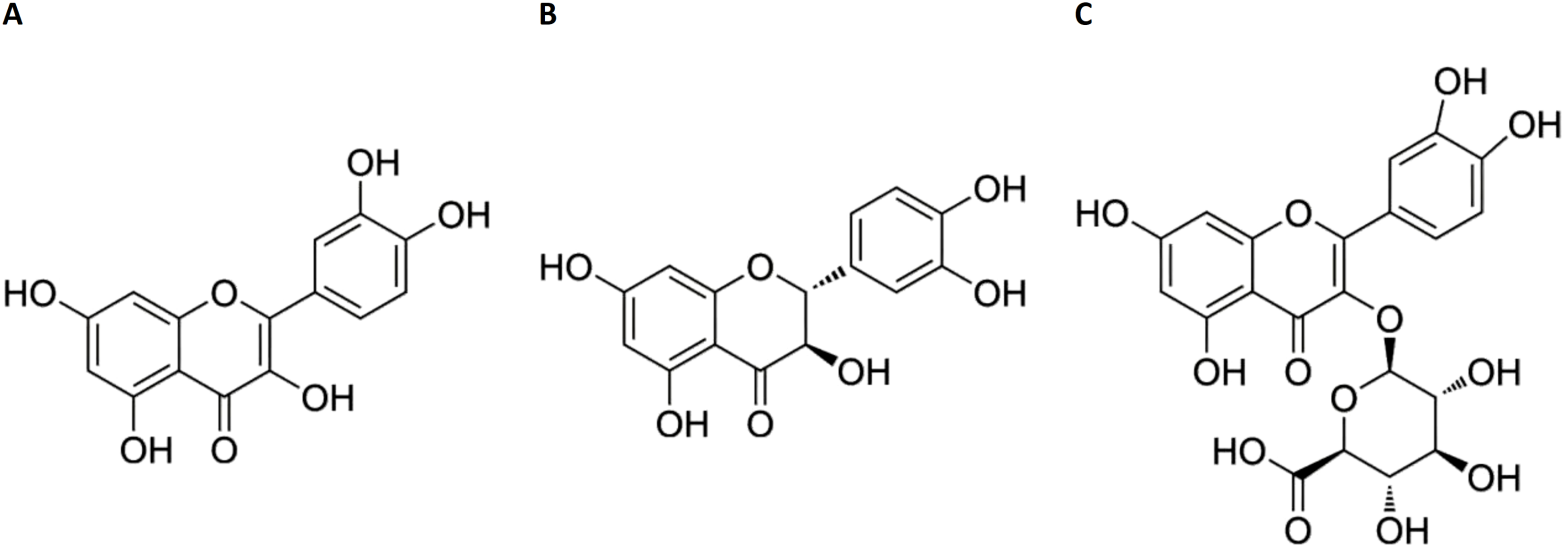
Chemical structures of quercetin **(A)** taxifolin **(B)** and miquelianin **(C)**.

Flavonoids, a class of secondary metabolites widely distributed in plants, exhibit immunoregulatory properties and therapeutic potential in inflammatory disorders (10). Notably, intestinal metabolism of flavonoids conjugated with amino acids generates deaminotyrosine, which protects against influenza infection by amplifying type I interferon (IFN) signaling pathways (11). Building on our previous discovery that isoquercitrin inhibits IAV and IBV virus replication (12), we selected three flavonoid derivatives for antiviral evaluation: quercetin (**Figure 1A**), taxifolin (**Figure 1B**), and miquelianin (**Figure 1C**). Quercetin, a polyphenol ubiquitously present in vegetative tissues (stems, leaves, buds) and reproductive organs (seeds, fruits, flowers) of angiosperms, exhibits pleiotropic bioactivities, including anti-inflammatory effects via cyclooxygenase-2 inhibition (13), antimicrobial activity against bacterial pathogens (13), suppression of metastatic cancer cell progression (14), and attenuation of acute lung injury (15). It demonstrates broad-spectrum antiviral efficacy against RNA viruses such as hepatitis C virus and human immunodeficiency virus (16), while synergistically enhancing neuraminidase inhibitors (e.g., oseltamivir and zanamivir) in influenza treatment (17). Taxifolin ((+)-dihydroquercetin), abundant in dietary sources (*Vitis vinifera, Allium cepa*), functions as a potent antioxidant through free radical scavenging (18), inhibits Coxsackievirus replication via direct interaction with the viral capsid protein (19), and suppresses tumorigenesis through modulation of the cyclic guanosine monophosphate–protein kinase G signaling pathway (20). Miquelianin (quercetin 3-O-glucuronide), characterized in medicinal plants (*Bupleurum chinense DC., Nelumbo nucifera Gaertn.*), reduces serum lipid levels via peroxisome proliferator-activated receptor gamma activation (21), enhances alveolar repair in pulmonary injury models (22), and mitigates IAV-induced pulmonary edema through stabilization of vascular endothelial cadherin (23).

This study systematically evaluated the *in vitro* and *in vivo* anti-influenza activities of quercetin, taxifolin, and miquelianin against IAV. Through integrated approaches, including Western blotting, reverse transcription-quantitative polymerase chain reaction (RT-qPCR), hematoxylin and eosin (H&E) staining, and immunohistochemical analysis, we demonstrated that miquelianin (6 mg/kg) significantly attenuated viral replication and host pathology. Network pharmacology analysis further identified key therapeutic targets (e.g., TAK1). These findings suggest that miquelianin could serve as a promising adjuvant or alternative therapeutic agent for influenza virus infections. Collectively, this work provides critical technical foundations for influenza prevention and control and reveals novel therapeutic targets and antiviral mechanisms for future drug development.

## 2 Materials and methods

### 2.1 Cells, virus, drugs, experimental animals, and antibodies

Fetal bovine serum (FBS, Gibco Lot.No.10438026), 10,000 U/mL penicillin–streptomycin solution, and Dulbecco’s modified Eagle’s culture medium (DMEM) were purchased from Thermo Fisher Scientific Corporation (Shanghai, China). Dimethyl sulfoxide (DMSO) was acquired from Sigma-Aldrich (Shanghai, China). Madin-Darby canine kidney (MDCK) cells are maintained at the Changchun Institute of Veterinary Science, Chinese Academy of Agricultural Sciences(Changchun, China). MDCK cell lines were cultured in DMEM supplemented with 10% FBS, 100 IU/mL penicillin, and 100 μg/mL streptomycin.

The H1N1-UI182 strain represents a mouse-adapted variant derived from the 2009 Influenza A H1N1 virus (A/Changchun/01/2009 (H1N1)), which has been serially propagated in MDCK cell cultures. The virus and cells were stored at the Changchun Institute of Veterinary Research, Chinese Academy of Agricultural Sciences (Changchun, China).

Quercetin (CAS No. 117-39-5; Cat NO. HY-18085), taxifolin (CAS No. 480-18-2; Cat NO. HY-N0136), miquelianin (CAS No. 22688-79-2; Cat NO. HY-13930) and baloxavir (Baloxavir acid; CAS No. 1985605-59-1; Cat NO. HY-109025A) were obtained from MedChemExpress (MCE, Shanghai, China). Balb/c female mice weighing 18 - 20 grams at 6 - 8 weeks of age were sourced from Beijing Vital River Laboratory Animal Technology Co., Ltd (Beijing, China).As directed by the manufacturer, the medication was dissolved in DMSO, diluted in DMEM with 2% FBS or phosphate butylamine saline or dissolved with DMSO before use, and diluted with 2% fetal bovine serum or butyl phosphate (PBS, pH 7.4) DMEM.

The antibodies against influenza virus nucleoprotein NP (ab20343, 1:1000) and β-actin (ab6272, 1:5000) were obtained from Abcam(Shanghai, China). Antibodies against IL-6 (12912, 1:1000), IL-1β(12703, 1:1000), p38 MAPK (8690S, 1:1000), TAK1 (5206S, 1:1000),p-TAK1 (9339S, 1:1000), JNK1 (3708S, 1:1000), JNK2 (9258S, 1:1000) and STAT3 (4904S, 1:1000) were obtained from Cell Signaling Technology(CST, Shanghai, China). Rat (A0216, 1:1000) and rabbit (A0208, 1:1000) secondary antibodies.

### 2.2 Cell culture, cytotoxicity, and virus infection assay

MDCK cells were cultured in DMEM containing 10% FBS, 100 IU/mL penicillin, and 100 μg/mL streptomycin at 37°C with 5% CO_2_. The cell supernatant was discarded when the cell density reached 70–80%, and then different concentrations of drugs (6.25, 12.5, 25, 50 and 100 μM) were inoculated in the cell plates, moreover 5 repeat holes were set for each concentration gradient. Virus-infected cells and non-infected cells were treated simultaneously with a working fluid at varying concentrations. Positive control wells (virus only) and negative control wells (without virus) were assigned to each plate. After 48 hours, cell viability was measured by CCK-8 assay using Cell Counting Kit-8 (C0039, Beyotime, Shanghai, China) following the manufacturer’s instructions. The absorbance at 450 nm for each pore was measured using an enzyme-linked immunosorbent plate reader, half inhibitory concentration (CC_50_) and half maximum effective concentration (EC_50_) value of cell viability were calculated by a nonlinear regression curve fitting analysis (GraphPad Prism 8.0) of cell viability to the log10 conversion concentration value. The SI was expressed as EC_50_/CC_50_.

### 2.3 Immunofluorescence staining

MDCK cells were seeded in 6-well plates and infected with H1N1-UI182 (MOI = 0.01) when they reached 70% confluence. One hour post-infection, quercetin (10 µM), taxifolin (50 µM), and miquelianin (100 µM) were added. After a 48-hour infection period, the cells were rinsed three times with phosphate-buffered saline (PBS, P1020, Solabio, China). Subsequently, the cells were fixed with 4% paraformaldehyde (room temperature) for 20 minutes, followed by permeabilization with 0.5% Triton X-100 (P0096, Beyotime, Shanghai, China) for 15 minutes. The cells were then blocked with 2% bovine serum albumin (BSA, 9048-46-8, Sigma, St. Louis, MO, USA) and incubated with the anti-influenza nucleocapsid protein primary antibody (ab104870, 1:500). After overnight incubation at 4°C, the cells were washed three times with PBS and incubated with secondary antibodies (goat anti-rabbit IgG H&L conjugated to AlexaFluor 488, ab150077, 1:500) for 2 hours in the dark. Finally, the cells were stained with Hoechst 33,258 (H3569, 1 µg/mL, Thermo Fisher Scientific, Shanghai, China) for 10 minutes, and the results were observed and recorded using a fluorescence microscope (APX100, Olympus, Shinjuku-ku, Tokyo, Japan).

### 2.4 Western blot

Cell or lung tissue samples were lysed with RIPA buffer (Beyotime, CAS: P0013B). The resulting supernatant was subsequently collected by centrifugation. Protein concentrations were quantified using the BCA Protein Quantitation Kit (Beyotime, CAS: P0009). After denaturation, the protein lysates were electrophoretically separated by SDS - PAGE and transferred onto PVDF membranes (Immobilon - P, IPVH00010).

The membranes were incubated in a 5% BSA solution at room temperature for one hour for blocking. Then, they were incubated with the primary antibody overnight at 4°C. After the primary antibody incubation, the membranes were incubated with the secondary antibody (Beyotime, China) for one hour at room temperature.

After each incubation period, the membranes were washed three times with TBST solution, with each wash lasting five minutes. After the final wash, proteins were detected using the chemiluminescent imaging system (Tanon, Jiading District, Shanghai, China). Meanwhile, ImageJ software (NIH, Bethesda, MD, USA) was used for grayscale analysis and quantification, with β-actin as the internal reference protein. Data visualization was performed using GraphPad Prism 8.0.

### 2.5 *In vivo* experiments in mice

Mice were randomly divided into six experimental groups (n=10 per group; 60 total animals): Control, Virus, Quercetin, Taxifolin, Miquelianin, and Baloxavir (positive control). The virus solution was diluted with PBS. The virus group and all treatment groups (quercetin, taxifolin, miquelianin, and baloxavir) received 50 µL of viral inoculum (5×MLD_50_) via intranasal instillation. After a 12-hour infection period, both Control and Virus groups were administered 100 µL PBS daily via intragastric gavage. Treatment groups received respective interventions for five consecutive days: the Quercetin group received oral quercetin (40 mg/kg/day), the Taxifolin group oral taxifolin (50 mg/kg/day), the Miquelianin group oral miquelianin (6 mg/kg/day), and the Baloxavir group subcutaneous baloxavir (5 mg/kg/day). Body weight and survival were monitored daily for 14 days post-infection. Two methods are used to determine death: physiological death and weight-based death (weight decline to below 75%). After extracting the eyes for blood collection, the mice are euthanized by cervical dislocation.

### 2.6 Pathological analysis

On day 5, three mice per group were randomly euthanized. Lung tissues were collected, weighed, and the lung index (lung weight (g)/body weight (g) × 100) was calculated to evaluate pathological changes. The lung index reflects the percentage of lung weight relative to final body weight. Tissues were fixed in 4% paraformaldehyde for 24–72 h, dehydrated through a graded ethanol series, embedded in paraffin, and sectioned into 4–8 µm slices. Sections were deparaffinized in xylene, rehydrated, and stained with H&E. After drying, slides were examined under a light microscope, and histopathological changes were assessed by blinded pathologists. Histopathological evaluation was performed to quantify lung injury severity. The scoring system ranged from 0 (no pathological alterations) to 4 (severe diffuse damage), based on H&E-stained lung sections assessed for five parameters: alveolar wall thickening, inflammatory infiltration and vascular congestion, parenchymal hemorrhage, bronchial epithelial necrosis, intra-alveolar cellular debris accumulation. The total pathology score was calculated as the sum of individual parameter scores. Data visualization was performed using GraphPad Prism 8.0.

### 2.7 Immunohistochemical assay

The sections were incubated with 0.3% hydrogen peroxide in methanol for 20 minutes at room temperature to quench endogenous peroxidase activity. After antigen retrieval, the tissue sections were blocked with 5% goat serum at room temperature for 20 minutes. After that, the sections were immunostained with an anti-A influenza virus antibody (NP,1:500) overnight at 4°C. Following rinsing, the tissue slides were exposed to a biotinylated goat anti-mouse secondary antibody (1:1000) for 1 hour at room temperature. The slides were stained with 3,3’-diaminobenzidine and counterstained with hematoxylin. Finally, the slides were evaluated using a microscope. The number of positive cells was quantified using ImageJ. Data visualization was performed using GraphPad Prism 8.0.

### 2.8 RNA isolation and quantitative RT-PCR

RNA extraction was performed using the HiPure Universal RNA Kit (Magen, China), followed by reverse transcription into cDNA using the PerfectReal Time kit (TAKARA, Japan). The RT-qPCR amplification reactions were then prepared. Primer sequences are provided in **Table 1**. Beta-actin served as the internal control gene for normalization.

**Table 1:** The sequence of primer for RT-qPCR.

Gene Name	Primer Sequence (5’ to 3’)
*β-actin*	F: 5’ -TGGAATCCCTGTGGGACCATGAAAC-3’
	R: 5’ -ATCATACTTGGCAGGTTTCTCCAGG-3’
*IL-6*	F: 5’ -AGTTGCCTTCTTGGGACTGATG-3’
	R: 5’ -GGGAGTGGTATCCTCTGTGAAGTCT-3’
*CXCL-10*	F: 5’- CAGCAGTCCGCAGTATAAACAGT-3’
	R: 5’- GCCAAGTACCTAACGCTCACC-3’
*IFN-α*	F: 5’ -GCACCCTGCCTCAGACTCAC-3’
	R: 5’ -TGCCTGGTCATCTCATGGAAG-3’
*IFN-γ*	F: 5’ -AGCCAAATCGTCTCCTTCTACTTC-3’
	R: 5’ -TGCACCTTGTTGCTGCTGTT-3’
*CCL-5*	F: 5’-CTCCTTGCTGCTTTGCCTAC-3’
	R: 5’-ACACACCTGGCGGTTCTTTC-3’

### 2.9 ELISA detection

Mice serum samples were detected using ELISA Kit (Elabscience, China) according to the manufacturer’s instructions. Serum expressions of IL-6(Cat NO. E-HSEL-M0003), CXCL10(Cat NO. E-EL-M0021),IFN-α(Cat NO. E-EL-M3054), IFN-γ(Cat NO. E-HSEL-M0007) and CCL5(Cat NO. E-EL-M0009) were detected.

### 2.10 EID_50_ detection

The EID_50_ (viral 50% embryo infectious dose) was determined using 9-day-old specific-pathogen-free (SPF) chicken embryos. Lung tissues from each experimental group were homogenized in OPTI-MEM medium supplemented with 400 U/mL penicillin-streptomycin using a mechanical homogenizer. Following centrifugation at 3,000 × g for 10 min, supernatants were serially diluted in 10-fold increments (10^-^¹ to 10^-8^). Each dilution was inoculated into three embryos (9-day-old SPF), which were incubated at 37°C for 48 h in a humidified atmosphere. Post-incubation, 50 μL of allantoic fluid from each embryo was mixed with an equal volume of 1% (v/v) chicken erythrocyte suspension and incubated at 25°C for 15 min. Hemagglutination activity was assessed visually, and viral titers were calculated via the Reed-Müench method, expressed as log_10_EID_50_/mL.

### 2.11 Network pharmacological identification of targets, functional enrichment and potential pathways

To acquire the structural information of miquelianin, the study utilized the NCBI PubChem database (https://pubchem.ncbi.nlm.nih.gov/). The simplified molecular input line entry system (SMILES) structural formula of miquelianin isomers was then imported into various target prediction databases, including Swiss Target Prediction (http://www.swisstargetprediction.ch), CTD (https://ctdbase.org/), SEA (https://sea.bkslab.org/), TargetNet (http://targetnet.scbdd.com/), ETCM (http://www.tcmip.cn/ETCM/index.php/Home/Index/), and Superpred (https://prediction.charite.de/subpages/target_prediction.php) to identify potential target proteins.

After merging the targets from these six databases, duplicates were removed, and the consolidated list of target proteins for miquelianin was harmonized into gene names through the UniProt database (https://www.uniprot.org/).

Using “Influenza” as a keyword, influenza-related genes were retrieved from three disease gene databases: GeneCards (https://www.genecards.org/), CTD (https://ctdbase.org/), and OMIM (https://www.omim.org/). The retrieved data were organized and entered as disease targets.

Miquelianin-related targets and influenza-related targets were identified using the online tool Venny (https://bioinfogp.cnb.csic.es/tools/venny/), and their intersection determined the effective therapeutic targets for miquelianin against influenza.

The STRING database (https://cn.string-db.org/) is an online bioinformatics resource for gene and protein interaction data. Targets were imported into STRING, filtered by an interaction score threshold of ≥0.4, and the resulting Protein-Protein Interaction (PPI) network and TSV files were downloaded and saved.

Cytoscape software (v3.9.0) was used to visualize the PPI network and construct a multidimensional network representing miquelianin-influenza interactions. Gene Ontology (GO) and Kyoto Encyclopedia of Genes and Genomes (KEGG) pathway enrichment analyses were performed on core targets using R software (v4.3.1) to explore the potential biological functions and key signaling pathways of miquelianin in influenza treatment. Statistical significance was determined with a q-value threshold of <0.05, and results were ranked in descending order of p-value magnitude to identify significantly enriched terms.

### 2.12 Statistical analysis

Statistical analyses were performed using one-way ANOVA. Quantitative data are presented as mean ± standard error (SE), and statistical significance was determined using GraphPad Prism^®^ 8.0 software. Differences were considered statistically significant at P < 0.05 compared to the Virus-infected group. Significance levels are denoted as *P<0.05, **P<0.01, and ***P<0.001.

## 3 Results

### 3.1 Taxifolin and miquelianin demonstrate inhibitory activity against IAV *in vitro*

**Figure 2:**
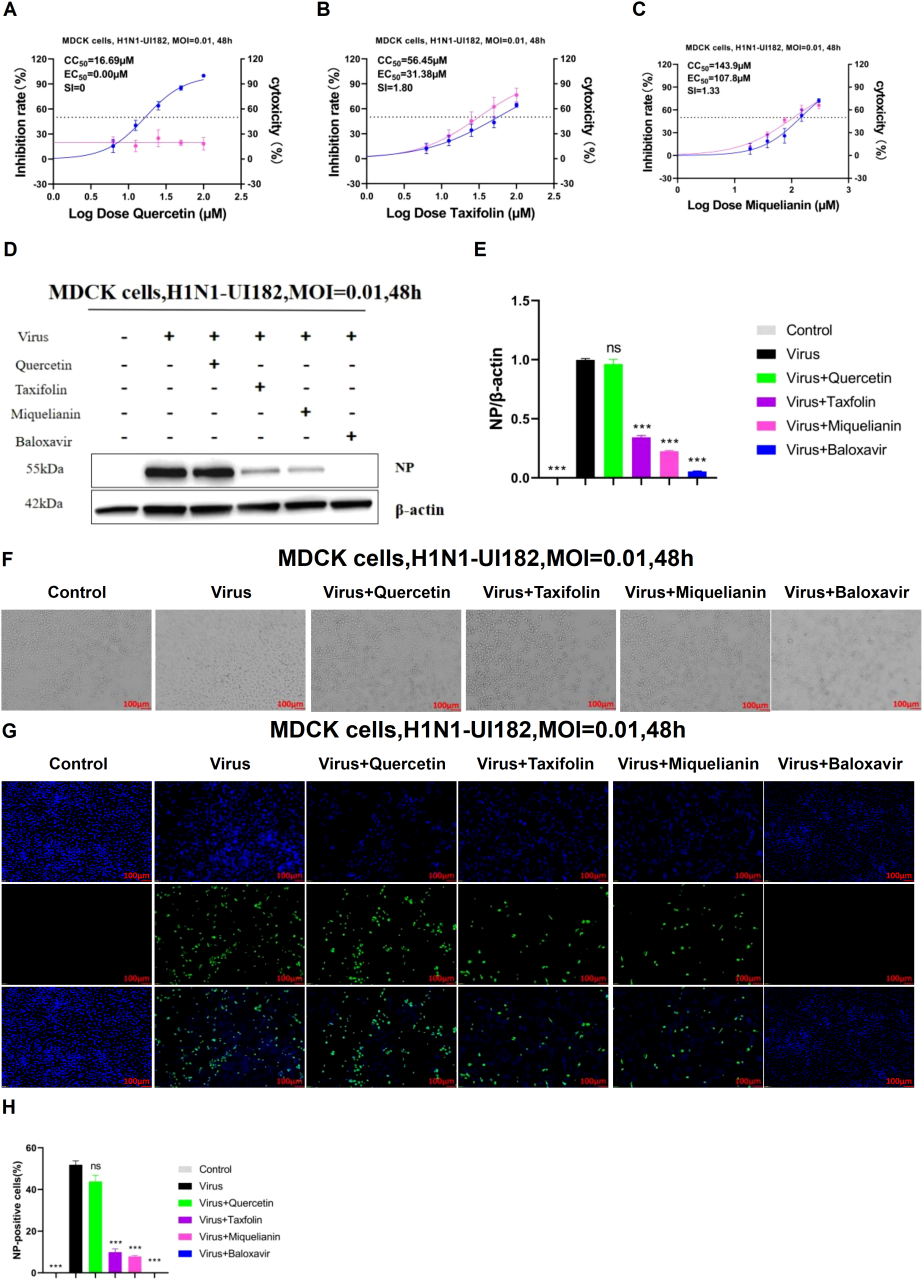
Inhibitory effects of quercetin, taxifolin and miquelianin on influenza A virus *in vitro*. **(A-C)** The inhibitory effects of quercetin, taxifolin, and miquelianin on influenza A virus in MDCK cells. As shown in the figure, the pink curve represents the dose-dependent relationship between the drug inhibition rate against the virus and dosage, while the blue curve represents the dose-dependent relationship between the drug’s cytotoxicity and dosage. Three independent experiments were conducted, and the figure shows the results of one representative experiment among the three performed. The EC50/IC50 values in the figure were obtained through curve fitting using GraphPad Prism 8.0 software and represent only the data from this experiment. The SI indicates the selection index (SI = CC50/EC50) of quercetin, taxifolin, and miquelianin. **(D)** After MDCK cells were infected with H1N1-UI182 at an MOI of 0.01, they were treated with quercetin (10 µM), taxifolin (50 µM), miquelianin (100 µM), and baloxavir (10 µM) for 48 h. The expression level of influenza NP protein was then analyzed by western blotting using β-actin as a loading control. **(E)** presents the quantitative analysis of **(D)**, performed using ImageJ software and GraphPad Prism 8.0 software. n=3; ***P<0.001. **(F)** The microscopic images of MDCK cells treated with quercetin (10 µM), taxifolin (50 µM), miquelianin (100 µM) and baloxavir (10 µM) at 48 h post-infection with H1N1-UI182. **(G)** After infection of MDCK cells with H1N1-UI182 at an MOI of 0.01, the cells were treated with quercetin (10 µM), taxifolin (50 µM), miquelianin (100 µM), and baloxavir (10 µM) for 48 h. Nuclei were stained with DAPI, and infected cells were detected by NP-specific immunofluorescence staining (scale bar: 100 µm). **(H)** The percentage of NP-positive cells in J was calculated, performed using ImageJ software and GraphPad Prism 8.0 software. n=3.

To evaluate the safe concentrations of quercetin, taxifolin, and miquelianin for sustained cell growth and their effective antiviral concentrations against H1N1-UI182, the CCK-8 assay was employed to measure the relative optical density (OD) of cells treated with serial drug dilutions for 48 hours. Results demonstrated that taxifolin and miquelianin significantly inhibited viral replication, whereas quercetin exhibited no detectable antiviral activity. Specifically, CC_50_ and EC_50_ values for quercetin were 16.96 µM and 0 µM, respectively, yielding a selectivity index (SI = CC_50_/EC_50_) of 0 (**Figure 2A**). For taxifolin, CC_50_ and EC_50_ values were 56.65 µM and 31.38 µM (SI = 1.8) (**Figure 2B**), while miquelianin showed CC_50_ and EC_50_ values of 143.9 µM and 107.8 µM (SI = 1.33) (**Figure 2C**).

To further assess the effects of the three compounds on influenza virus NP expression, MDCK cells were cultured in 6-well plates. When cell confluence reached 70–80%, the supernatant was aspirated, and cells were infected with viral inoculum (MOI=0.01). At 1 hour post-infection (hpi), quercetin (10 µM), taxifolin (50 µM), miquelianin (100 µM), and baloxavir (10 µM) were added to designated wells. After 48 hours, WB and IF imaging were performed in parallel to evaluate viral NP levels and antigen distribution. WB analyses demonstrated significantly elevated NP expression in the virus-infected group compared to the negative control, while taxifolin and miquelianin treatments markedly reduced NP expression. In contrast, quercetin showed no substantial reduction in NP expression (**Figures 2D, E**). Cell morphology was observed under microscopy (**Figure 2F**), revealing severe cytopathic effects in the virus-infected group, characterized by increased intercellular gaps and widespread cell detachment. Taxifolin and miquelianin treatments significantly improved cell morphology and viability. IF results indicated that taxifolin and miquelianin significantly reduced the proportion of NP-positive cells (P < 0.05 vs. virus group), whereas quercetin exhibited no statistically significant effect (**Figures 2J, K**). Collectively, these findings demonstrate that taxifolin and miquelianin effectively suppress IAV replication *in vitro*, highlighting their potent anti-IAV activity.

### 3.2 Taxifolin and miquelianin confer protection against IAV lethal-infection in mice

To evaluate the therapeutic efficacy of quercetin, taxifolin, and miquelianin against IAV, we utilized a lethal H1N1-UI182 infectious mouse model. Weight loss and mortality in infected mice were monitored over a 14-day period, followed by statistical analysis.

**Figure 3:**
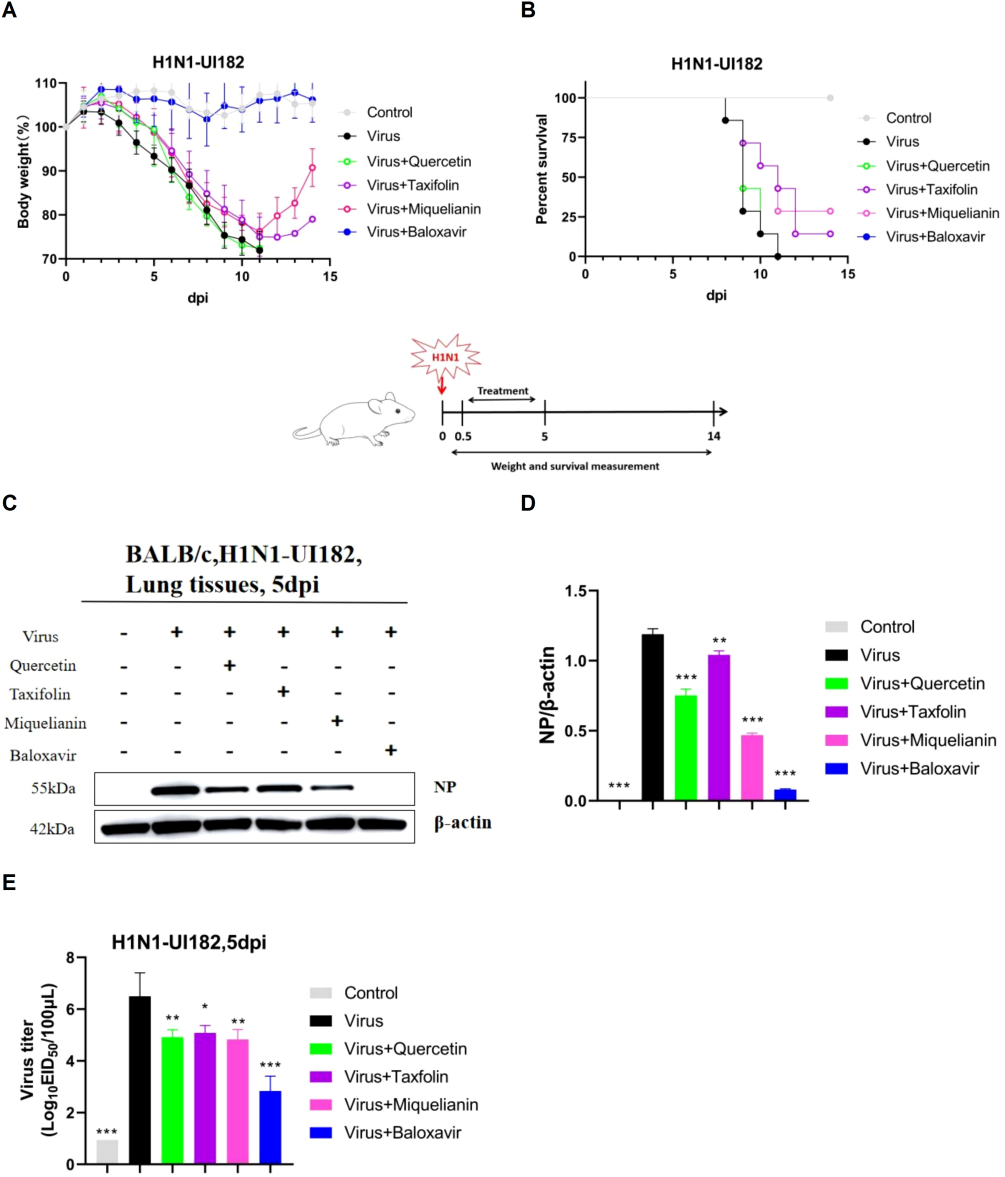
Antiviral effects of quercetin, taxifolin, and miquelianin against H1N1-UI182 in a mouse model. **(A)** Body weight changes in control group (PBS, oral administration), virus-infected group (PBS, oral administration), quercetin treatment group (40 mg/kg, oral administration), taxifolin treatment group (50 mg/kg, oral administration), miquelianin treatment group (6 mg/kg, oral administration), and baloxavir group (5 mg/kg, subcutaneous injection). **(B)** Survival rate of H1N1-UI182-infected mice. **(C)** Western blot analysis of NP expression levels in mouse lung tissues at 5 dpi, with β-actin serving as a loading control. **(D)** Quantitative analysis of viral NP protein expression using ImageJ and GraphPad Prism 8.0 software.n=3; **P<0.01; ***P<0.001. **(E)** Effects of quercetin, taxifolin, and miquelianin phosphate on viral titers in mouse lungs at 5 dpi.n=3; *P<0.05; **P<0.01; ***P<0.001.

The results demonstrated that mice experienced significant weight loss beginning on day 3 post-infection. In contrast, mice in the taxifolin- and miquelianin-treated groups began to regain weight starting on day 12 (**Figure 3A**). All mice in the virus control group succumbed to infection by day 9, whereas survival rates in the taxifolin- and miquelianin-treated groups significantly improved to 14.3% and 28.6%, respectively, compared to the virus control group (**Figure 3B**). Notably, the quercetin-treated group exhibited 100% mortality by day 9.

WB analysis revealed substantial upregulation of nucleoprotein (NP) expression in the lung tissues of the virus control group. However, treatment with quercetin, taxifolin, and miquelianin effectively reduced NP expression levels in lung tissues (**Figures 3C, D**). EID_50_ measured at 5 days post-infection demonstrated significant reductions in viral titers across all treatment groups compared to the virus control group (**Figure 3E**). These results indicate that taxifolin and miquelianin confer protection against lethal IAV infection in mice.

### 3.3 Taxifolin and miquelianin ameliorate pulmonary pathological damage in mice lethally infected with IAV

Influenza virus infection induces a range of lung injuries in the host. To explore potential interventions, we investigated the therapeutic effects of quercetin, taxifolin, and miquelianin on influenza-associated pulmonary pathology.

**Figure 4:**
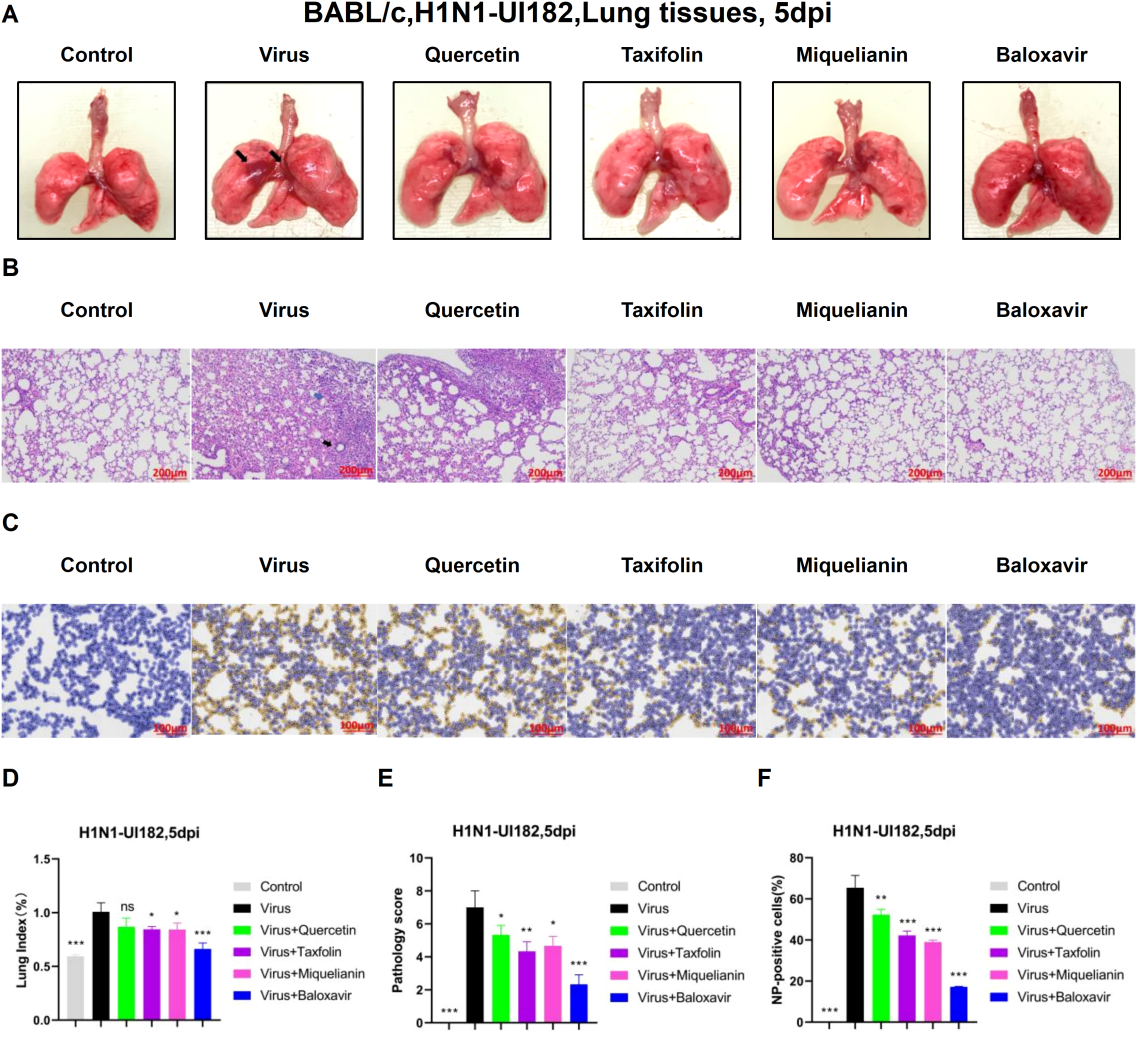
The effect of quercetin, taxifolin, and miquelianin on H1N1-UI182-induced pathological damage. **(A)** Gross lung morphology (The black arrows indicate extensive hemorrhagic lesions with associated surface damage). **(B)** H&E staining of lung tissues (The black arrow indicates lymphocytic and neutrophilic infiltration, whereas the blue arrow denotes eosinophilic accumulation within alveolar spaces). **(C)** Immunohistochemical staining for viral NP. **(D)** Lung index. n=3; *P<0.05; ***P<0.001. **(E)** Lung pathology scoring. n=3; *P<0.05; **P<0.01; ***P<0.001. **(F)** Quantification of NP-positive cells in lung tissues analyzed using ImageJ and GraphPad Prism 8.0 software. n=3; **P<0.01; ***P<0.001.

Morphological analysis revealed extensive hemorrhagic lesions and surface damage (black arrows) in lung tissues from the virus-infected group. Both taxifolin and miquelianin treatments significantly ameliorated these pathological manifestations (**Figure 4A**) and reduced the lung index (**Figure 4D**). H&E staining of lung tissues demonstrated that influenza virus infection induced alveolar wall thickening, necrotic cell debris, infiltration of lymphocytes and neutrophils (black arrows) and eosinophilic effusion within alveolar spaces (blue arrows). These pathological alterations were markedly attenuated by taxifolin and miquelianin (**Figures 4B, E**). Immunohistochemical analysis of viral nucleoprotein (NP) revealed that taxifolin and miquelianin significantly suppressed influenza virus NP expression in lung tissues (**Figures 4C, F**). Collectively, these results demonstrate that taxifolin and miquelianin inhibit viral protein expression and mitigate influenza virus-induced pathological damage in major organs of mice.

### 3.4 Quercetin, taxifolin, and miquelianin inhibit cytokines induced by IAV

**Figure 5:**
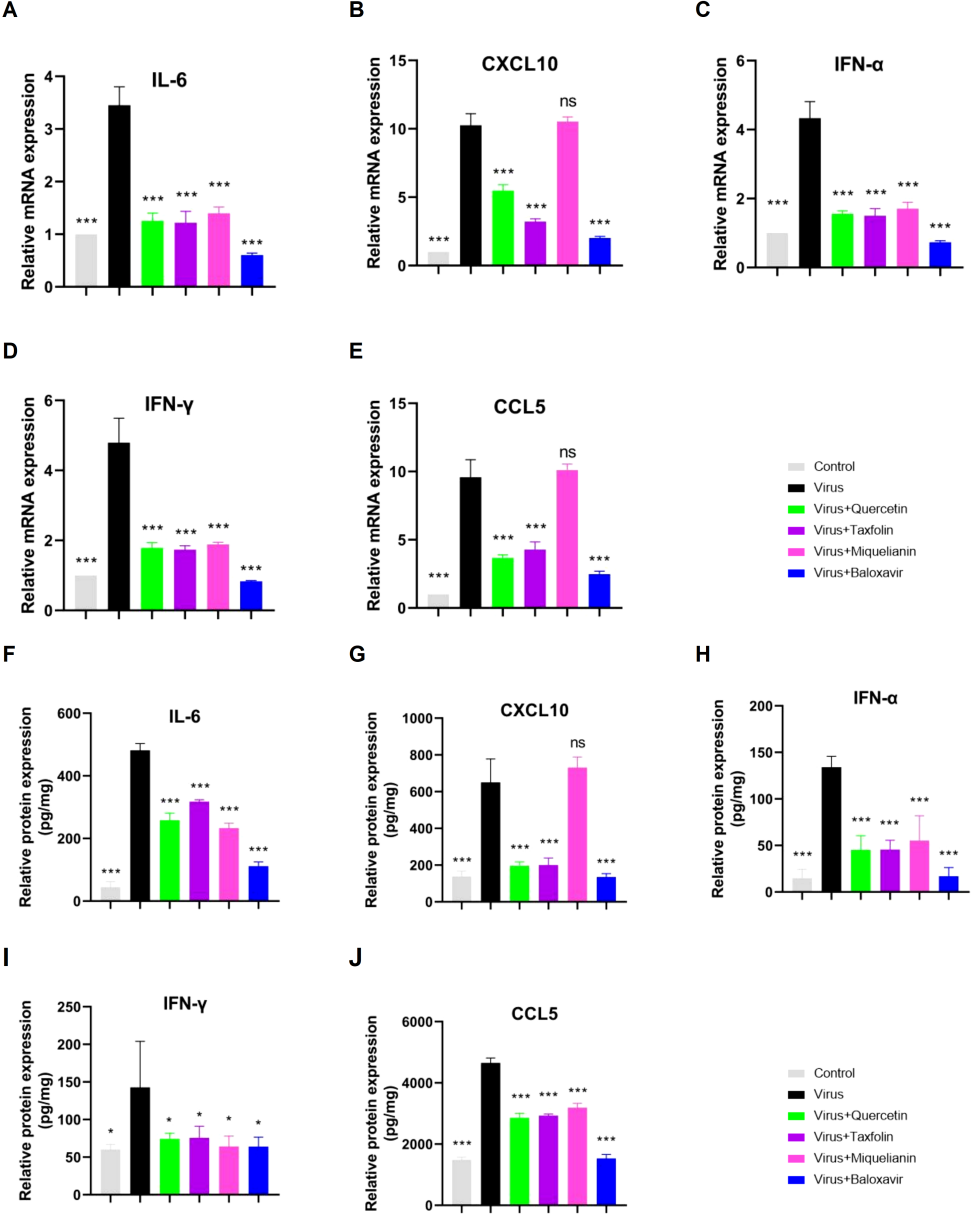
Effects of quercetin, taxifolin, and miquelianin on cytokine expression. **(A–E)** mRNA levels of proinflammatory cytokines in mouse lung tissue quantified by qRT-PCR: **(A)** IL-6, **(B)** CXCL10, **(C)** IFN-α, **(D)** IFN-γ, and **(E)** CCL5. **(F–J)** Protein levels of corresponding cytokines assessed by ELISA: **(F)** IL-6, **(G)** CXCL10, **(H)** IFN-α, **(I)** IFN-γ, and **(J)** CCL5. Data analysis was performed using GraphPad Prism 8.0 software. n=3; *P<0.05; **P<0.01; ***P<0.001.

Viral infections often hijack the host immune system, leading to abnormal immune responses, promoting the release and accumulation of inflammatory cytokines, and ultimately triggering a ‘cytokine storm, which is a major factor contributing to poor prognosis in infected individuals (24). In this study, we detected the mRNA and protein levels of IL-6 (**Figure 5A**), CXCL10 (**Figure 5B**), IFN-α (**Figure 5C**), IFN-γ (**Figure 5D**) and CCL5 (**Figure 5E**) in the lung tissues of IAV-infected mice using RT-qPCR. The results demonstrated that treatment with quercetin, taxifolin, and miquelianin significantly reduced the expression of these cytokines in the lung tissues of mice. Furthermore, ELISA analysis of the protein levels of IL-6 (**Figure 5F**), CXCL10 (**Figure 5G**), IFN-α (**Figure 5H**), IFN-γ (**Figure 5I**) and CCL5 (**Figure 5J**) revealed similar results, indicating that quercetin, taxifolin, and miquelianin effectively inhibit cytokine production induced by IAV infection.

### 3.5 Miquelianin effectively shielded mice from lethal infection and pulmonary pathological damage induced by IAV, demonstrating a dose-dependent protective effect

**Figure 6:**
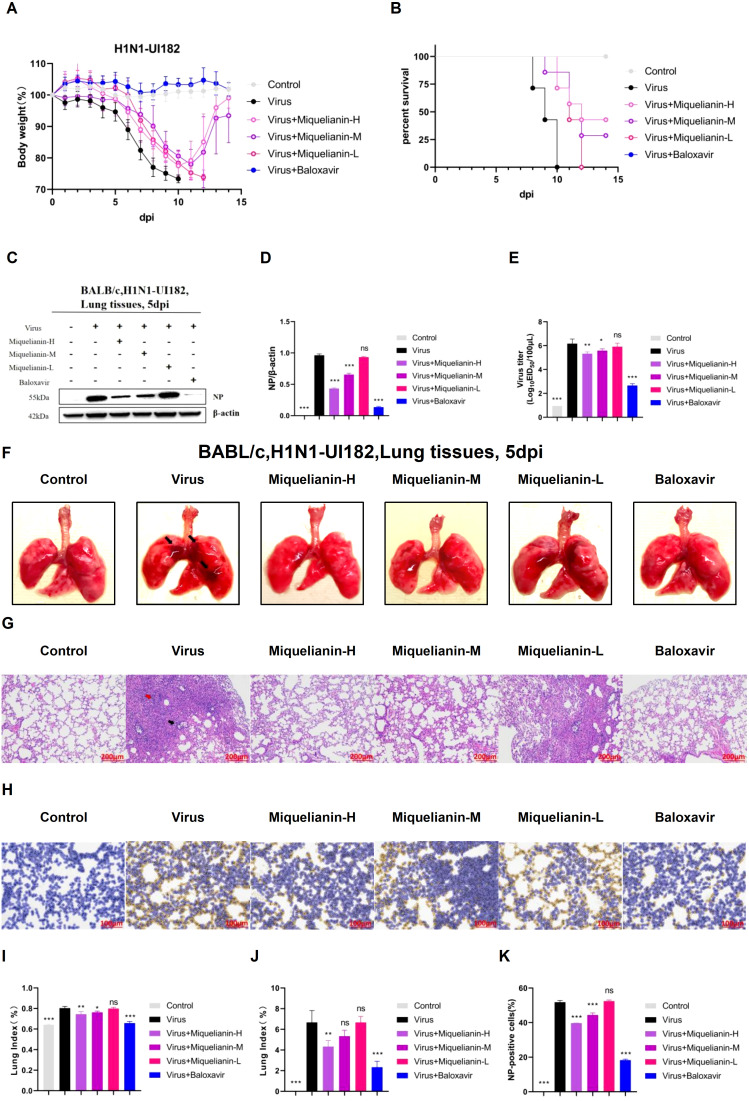
Antiviral effects of miquelianin-H, miquelianin-M, and miquelianin-L against H1N1-UI182 in a mouse model. **(A)** Body weight changes in control group (PBS, oral administration), virus-infected group (PBS, oral administration), miquelianin-H group (12 mg/kg, oral administration), miquelianin-M group (6 mg/kg, oral administration), miquelianin-L group (3 mg/kg, oral administration), and baloxavir group (5 mg/kg, subcutaneous injection). **(B)** Survival rate of H1N1-UI182-infected mice. **(C)** Western blot analysis of viral NP expression in lung tissues at 5 days post-infection (dpi), with β-actin as a loading control **(D)** Quantification of NP-positive cells in lung tissues.n=3; ***P<0.001. **(E)** Effects of miquelianin at different doses on viral titers in mouse lungs at 5 dpi. n=3; *P<0.05; **P<0.01; ***P<0.001. **(F)** Gross lung morphology (The black arrows indicate extensive hemorrhagic lesions with associated surface damage). **(G)** H&E staining of lung tissues (The black arrow indicates neutrophil infiltration, whereas the red arrow denotes necrotic cellular debris within the bronchiolar lumens). **(H)** Immunohistochemical staining for viral NP. **(I)** Lung index. n=3; *P<0.05; **P<0.01; ***P<0.001. **(J)** Lung pathology scoring.n=3; **P<0.01; ***P<0.001. **(K)** Quantitative analysis of NP-positive cells in lung tissues. Data analysis was performed using ImageJ and GraphPad Prism 8.0 software. n=3; ***P<0.001.

Our previous studies demonstrated that miquelianin significantly improved survival rates in IAV-infected mice. To determine its optimal therapeutic dose *in vivo*, three treatment groups were established: high-dose (12 mg/kg/day), medium-dose (6 mg/kg/day), and low-dose (3 mg/kg/day) miquelianin. By day 11 post-infection, mice in the treatment groups began to regain body weight (**Figure 6A**). All mice in the virus control group succumbed by day 10, whereas survival rates in the high- and medium-dose groups significantly increased to 42.9% and 28.6%, respectively (**Figure 6B**). The low-dose group delayed mortality, with all mice succumbing by day 11.

Western blot analysis revealed elevated viral NP expression in infected lungs, which was attenuated in both high- and medium-dose miquelianin-treated groups (**Figures 6C, D**). The EID_50_ measured at 5 days post-infection showed significant viral titer reductions in high- and medium-dose groups compared to the virus control (**Figure 6E**).

Morphological analysis of lung tissues from the virus-infected group revealed extensive hemorrhagic lesions and surface damage (black arrows), which were ameliorated by high- and medium-dose treatments (**Figure 6F**). The lung index was also significantly reduced in these groups (**Figure 6I**). H&E staining demonstrated IAV-induced alveolar wall thickening, necrotic cell debris in bronchiolar lumens (red arrow), and neutrophil infiltration (black arrow). These pathologies were markedly attenuated by high- and medium-dose miquelianin (**Figures 6G, J**).

Immunohistochemical analysis confirmed that high- and medium-dose miquelianin significantly suppressed viral NP expression in lung tissues (**Figures 6H, K**). Collectively, these results demonstrate that miquelianin effectively protects mice from IAV-induced lethal infection and pulmonary pathology in a dose-dependent manner.

### 3.6 Miquelianin enhances the protective efficacy in the H1N1-UI182-infected mouse model through the activation of the MAPK signaling pathway

**Figure 7:**
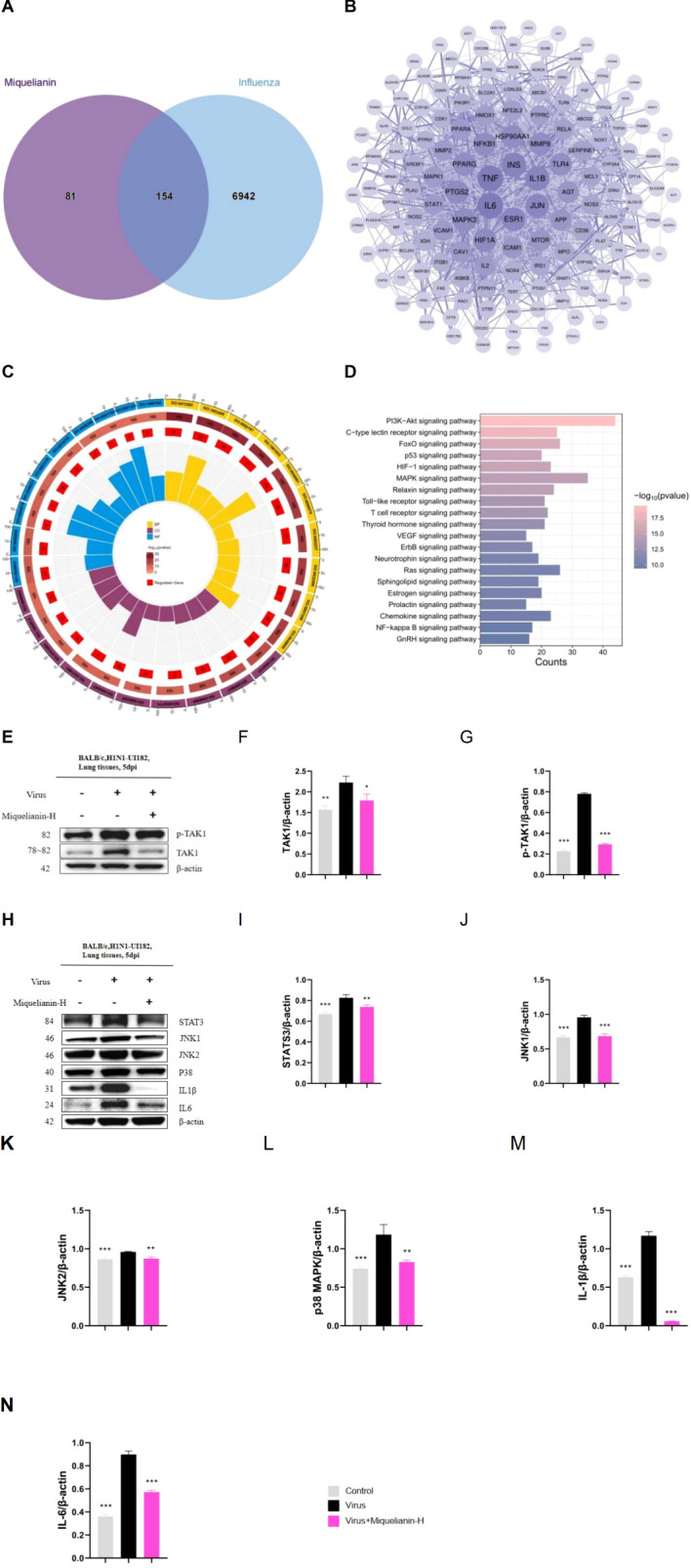
Network pharmacology and mechanism of Miquelianin. **(A)**The Venn diagram of core genes in Miquelianin target genes and influenza target genes. **(B)** The PPI network diagram of the interaction among the 154 overlapping genes. **(C)** GO pathway enrichment analysis. **(D)** KEGG pathway analyses of the 154 overlapping genes. **(E-H)** Western blot images of **(E-G)** TAK1, p-TAK1, **(H-N)** STAT3,JNK1, JNK2, p38 MAPK,IL - 1β, IL-6. The quantification of Western blot bands is shown. Data analysis was performed using GraphPad Prism 8.0 software. n=3; *P<0.05; **P<0.01; ***P<0.001.

The Swiss Target Prediction, CTD, SEA, TargetNet, ETCM, and SuperPred databases identified 235 target points associated with Miquelianin. Subsequent queries in the GeneCards, CTD, and OMIM databases yielded 1604, 5938, and 422 influenza-related target points, respectively. Intersection analysis with the target genes of miquelianin uncovered a final set of 154 potential therapeutic target points associated with influenza (**Figure 7A**). These 154 candidates were imported into the STRING database and visualized using Cytoscape software (version 3.9.0) as part of the PPI network, with a filtering criterion of ‘minimum required interaction score ≥ 0.4’ (**Figure 7B**). Enrichment analyses using the GO and KEGG revealed the principal biological processes of the identified target proteins. A GO and KEGG pathway enrichment analysis was performed on the 154 potential influenza-related target points using R software. Utilizing the R package ‘clusterProfiler,’ GO enrichment analysis yielded a total of 2561 entries, including 2258 associated with biological processes (BP), such as reactive oxygen species metabolism and biosynthesis, cellular response to chemical stress and peptides, and positive regulation of smooth muscle cell proliferation; 203 entries related to molecular function (MF), including heme and tetrapyrrole binding, nuclear receptor activity, ligand-activated transcription factor activity, and phosphoprotein binding; and 100 entries associated with cellular components (CC), such as membrane rafts, microdomains, secretory granule lumens, and cytoplasmic vesicle lumens. Significant enriched results were identified based on P-values. The top 10 entries in BP, MF, and CC were visualized (**Figure 6C**). The analysis indicates that potential targets are involved in biological functions such as inflammatory response and cell cycle regulation, which are closely associated with influenza pathogenesis. This suggests that Miquelianin’s therapeutic efficacy against influenza is multifaceted, involving multiple biological processes.

We performed KEGG pathway enrichment analysis on the 154 target genes that intersect with those associated with Miquelianin’s antiviral activity against influenza using the R language’s ‘ClusterProfiler’ package. This analysis identified 160 signal transduction pathways relevant to Miquelianin’s antiviral intervention, with a false discovery rate (p-value) threshold of <0.05. The pathways primarily included the HIF-1 signaling pathway, TNF signaling pathway, IL-17 signaling pathway, PI3K-Akt signaling pathway, Neurotrophin signaling pathway, Sphingolipid signaling pathway, MAPK signaling pathway, Prolactin signaling pathway, NOD-like receptor signaling pathway, and cAMP signaling pathway. The enriched pathways were ranked based on P-value in descending order, and the top 20 pathways were visualized in **Figure 6D**.

Based on reviewing existing related research, the mitogen-activated protein kinase (MAPK) signaling pathway has been shown to be closely associated with inflammatory reactions. Therefore, we selected MAPK to investigate its involvement in the regulation of IAV and miquelianin. We conducted WB analysis of TAK1, p-TAK1, STAT3, JNK1, JNK2, p38 MAPK, IL-1β, and IL-6 (**Figures 6E–N**). The results demonstrated that IAV infection promoted the phosphorylation of TAK1 and induced the expression of JNK1, p38 MAPK, as well as their downstream signaling pathways, including STAT3, IL-1β, and IL-6. However, treatment with miquelianin reversed these changes. These findings collectively indicate that TAK1 is a potential molecular target of miquelianin. Mechanistically, miquelianin likely exerts its anti-influenza activity against IAV through dual suppression of the TAK1-dependent JNK1 and p38 MAPK signaling cascades.

## 4 Discussion

The high genetic variability of influenza viruses facilitates rapid mutation of surface antigens, resulting in impaired immune recognition and subsequent widespread infections. Rising resistance to conventional antiviral agents further highlights the urgent requirement for novel influenza therapeutics (9). Natural plant extracts have gained prominence as a rich repository of lead compounds in drug discovery. Among phytochemical constituents—including phenolic compounds, alkaloids, polysaccharides, and flavonoids—the latter have been extensively investigated for their therapeutic potential. To date, over 9,000 flavonoid compounds have been identified across plant taxa, categorized into major subclasses such as flavones, flavonols, and isoflavones. These molecules have garnered considerable research interest owing to their pleiotropic biological activities (25), notably antioxidant (26) and anti-inflammatory effects (27). In this study, we systematically evaluated the anti-influenza activity of quercetin, taxifolin, and miquelianin against influenza A virus (IAV) through integrated *in vitro* and *in vivo* approaches. Notably, miquelianin demonstrated potent inhibition of IAV replication in both experimental models.

Influenza virus infection triggers an aberrant immune response characterized by excessive cytokine production, commonly referred to as cytokine release syndrome or cytokine storm. This phenomenon involves severe inflammation coupled with inadequate anti-inflammatory regulation (28), disrupting immune homeostasis (24). During early infection, viral particles migrate from the upper to lower respiratory tract through respiratory mechanics, reaching the pharynx and larynx. This facilitates infection of lung epithelial cells and alveolar macrophages, leading to the activation of inflammatory and antiviral signaling pathways (29). Although inflammation is essential for host defense against IAV, it paradoxically contributes to adverse clinical outcomes, including organ injury and mortality. The robust inflammatory cascade induced by IAV drives lung epithelial damage, acute pulmonary inflammation, fever, and fibrotic remodeling. Elevated levels of early-response cytokines (IL-6, IFN-α, IFN-γ) and chemokines (CCL5, CXCL10) are strongly associated with symptom severity (Nara et al., 2015). Our data demonstrate that miquelianin treatment significantly attenuates IAV-induced cytokine expression (IL-6, IFN-α, IFN-γ) in lethally infected mice, improving survival rates and ameliorating lung histopathology.

KEGG pathway analysis within the network pharmacology framework revealed that the anti-influenza mechanism of miquelianin against IAV may involve modulation of the MAPK signaling pathway. MAPK signaling pathway comprises three principal branches: ERK, JNK and p38 MAPK. These pathways exhibit dual roles in IAV infection by modulating host cell signal transduction—promoting viral replication while simultaneously mediating immune responses. Specifically, ERK activation facilitates IAV endocytosis and endosomal acidification, enabling viral genome release (30); p38 MAPK activation drives inflammatory cytokine production (e.g., IL-6), exacerbating host tissue damage while paradoxically enhancing viral replication via phosphorylation of viral proteins such as NS1 (31); JNK activation induces host stress granule formation to restrict viral mRNA translation, though IAV counteracts this defense by suppressing JNK activity through its NS1 protein (32). Consequently, MAPK pathway inhibition may suppress IAV replication (33). As a critical upstream regulator of MAPK signaling, transforming growth factor-β-activated kinase 1 (TAK1)—a member of the MAP3K (MAPK kinase kinase) family—activates JNK and p38 MAPK pathways by phosphorylating downstream MKKs (MAPK kinases, including MKK4/7 and MKK3/6) (34). TAK1 activation is typically triggered by cellular stressors (e.g., inflammatory cytokines, oxidative stress) or pattern recognition receptors (e.g., TLRs, IL-1R), ultimately governing cellular processes such as proliferation, apoptosis, inflammation, and immune regulation (35). Based on this regulatory network, we hypothesize that miquelianin attenuates IAV-induced inflammation by inhibiting TAK1 overactivation, thereby suppressing downstream JNK1 and p38 MAPK signaling, reducing cytokine release, and mitigating inflammatory tissue damage.

WB analysis demonstrated that miquelianin inhibits the phosphorylation of TAK1 overactivation, thereby suppressing both JNK1 and p38 MAPK activation and subsequently reducing the expression of IL-6, IL-1β, and STAT3. These findings collectively indicate that TAK1 is a potential molecular target of miquelianin. Mechanistically, miquelianin likely exerts its anti-influenza activity against IAV through dual suppression of the TAK1-dependent JNK1 and p38 MAPK signaling cascades.

Dosage is a critical determinant of pharmacological efficacy. We therefore evaluated the dose-response relationship of miquelianin against influenza A H1N1-UI182 strain. Miquelianin exhibited concentration-dependent suppression of H1N1-UI182 replication *in vitro*. While low-dose miquelianin failed to improve survival rates in H1N1-UI182-infected mice, it significantly extended median survival time compared to untreated controls.

This study has several limitations. First, while the observed suppression of TAK1 levels in both *in vitro* and *in vivo* models may partially account for miquelianin’s anti-IAV activity, its antiviral mechanism likely involves dual effects—direct inhibition of viral replication and indirect modulation of TAK1 signaling. Furthermore, the pharmacokinetic properties of miquelianin (e.g., absorption, metabolism, and excretion) remain uncharacterized, which will be a priority for future investigations. Second, although the drug concentrations used here were based on precedent studies, additional dose-response experiments are required to identify optimal therapeutic thresholds. Finally, while our data suggest TAK1 as a potential intersectional node between miquelianin and IAV pathogenesis, conclusive validation necessitates mechanistic studies using TAK1-knockout models or selective inhibitors to delineate its precise role.

## 5 Conclusions

Miquelianin demonstrates robust *in vitro* and *in vivo* antiviral activity against IAV. It effectively inhibits viral replication and significantly suppresses the excessive production of virus-induced cytokines through the TAK1/JNK1 and TAK1/p38 MAPK signaling pathways. Additionally, it reduces lung tissue damage, suggesting its potential as a candidate therapeutic agent for influenza virus infection.

## Data Availability

The original contributions presented in the study are included in the article/supplementary material. Further inquiries can be directed to the corresponding authors.
